# Field assessment of balance in 10 to 14 year old children, reproducibility and validity of the Nintendo Wii board

**DOI:** 10.1186/1471-2431-14-144

**Published:** 2014-06-10

**Authors:** Lisbeth Runge Larsen, Martin Grønbech Jørgensen, Tina Junge, Birgit Juul-Kristensen, Niels Wedderkopp

**Affiliations:** 1 Institute of Regional Health Services Research, University of Southern Denmark, Winsloewparken 193, Odense C 5000, Denmark; 2 Department of Geriatrics, Aalborg University Hospital, Hobrovej 18-22, Aalborg 9000, Denmark; 3 Institute of Sports Science and Clinical Biomechanics, University of Southern Denmark, Campusvej 55, Odense M 5230, Denmark; 4 Sports Medicine Clinic, Orthopaedic Deparntment Hospital of Lillebaelt, 5500 Middelfart, Vejle 7100, Denmark; 5 Department of Health Sciences, Institute of Occupational Therapy, Physiotherapy and Radiography, Bergen University College, Bergen 5009, Norway

**Keywords:** Sway, Children, Nintendo Wii, Reproducibility of results, Validity

## Abstract

**Background:**

Because body proportions in childhood are different to those in adulthood, children have a relatively higher centre of mass location. This biomechanical difference and the fact that children’s movements have not yet fully matured result in different sway performances in children and adults. When assessing static balance, it is essential to use objective, sensitive tools, and these types of measurement have previously been performed in laboratory settings. However, the emergence of technologies like the Nintendo Wii Board (NWB) might allow balance assessment in field settings. As the NWB has only been validated and tested for reproducibility in adults, the purpose of this study was to examine reproducibility and validity of the NWB in a field setting, in a population of children.

**Methods:**

Fifty-four 10–14 year-olds from the CHAMPS-Study DK performed four different balance tests: bilateral stance with eyes open (1), unilateral stance on dominant (2) and non-dominant leg (3) with eyes open, and bilateral stance with eyes closed (4). Three rounds of the four tests were completed with the NWB and with a force platform (AMTI). To assess reproducibility, an intra-day test-retest design was applied with a two-hour break between sessions.

**Results:**

Bland-Altman plots supplemented by Minimum Detectable Change (MDC) and concordance correlation coefficient (CCC) demonstrated satisfactory reproducibility for the NWB and the AMTI (MDC: 26.3-28.2%, CCC: 0.76-0.86) using Centre Of Pressure path Length as measurement parameter. Bland-Altman plots demonstrated satisfactory concurrent validity between the NWB and the AMTI, supplemented by satisfactory CCC in all tests (CCC: 0.74-0.87). The ranges of the limits of agreement in the validity study were comparable to the limits of agreement of the reproducibility study.

**Conclusion:**

Both NWB and AMTI have satisfactory reproducibility for testing static balance in a population of children. Concurrent validity of NWB compared with AMTI was satisfactory. Furthermore, the results from the concurrent validity study were comparable to the reproducibility results of the NWB and the AMTI. Thus, NWB has the potential to replace the AMTI in field settings in studies including children. Future studies are needed to examine intra-subject variability and to test the predictive validity of NWB.

## Background

Regardless of one’s age, an adequate balance control is an important ability in relation to coping with daily activities, participating in sport activities and avoiding sport injuries [1-5].

Standing balance control in children, measured as sway performance, differs from sway performance in adults [6]. Body size proportions in children and adults differ, and the “top-heaviness” of children results in a relatively higher centre of mass location. Along with the fact that children’s movements have not yet fully matured, the result is different sway performances in younger children (below the age of 10) and adults, in terms of both amplitude and velocity of sway (temporal, spatial and continuous refinements of postural strategies) [6-9]. The refinement of postural control strategy continues beyond 10 years of age, probably until young adult age [7].

The use of a force platform to assess standing balance control, as Centre Of Pressure path Length (COPL) excursions, or Centre Of Pressure (COP) velocity, is frequent in laboratory settings [10-14] but not in field settings. Outcome measures obtained with a force platform are objective and previously considered a ‘gold standard’ for assessing standing balance [15], as the method is capable of quantifying subtle changes, that are otherwise difficult to quantify using subjective outcomes [5]. Furthermore, the force platform technique provides clinicians and researchers with a valuable ‘bio-signature’ similar to that seen in gait analyses and potentially capable of predicting injuries or fall accidents [1-5]. However, force platforms are often advanced to operate, economic costs are high, feasibility is low and equipment is difficult to transport. Thus there is a need for feasible, low-cost equipment for reliable and valid measurement of sway performance in both laboratory and field settings.

Satisfactory test-retest reproducibility in the laboratory does not necessarily result in satisfactory reproducibility in the field. Reproducibility can be disturbed by noise, visual disturbances and difficulties in concentration in a noisy and uncontrolled environment. To our knowledge, the reproducibility of the NWB and the AMTI has not been examined in children in a field setting. If test-retest reproducibility is inadequate, the validity of the equipment to measure sway will be further affected by large measurement variations between the measuring devices.

It has recently been suggested that the COPL balance measures extracted from the low-cost Nintendo Wii board (NWB) are both reproducible (intra-class correlation (ICC) values of 0.79-0.94) and comparable with sway measures obtained from laboratory force platforms (ICC of 0.77-0.89) [13,15-18] for the measurement of undisturbed standing balance of young and older adults in a laboratory setting. Measurement in children could result in larger variations and poorer test-retest performance, however, due to lesser motor development and reduced postural control in children, as well as difficulties in concentrating on the tests and the instructions. Furthermore, children have lower weight than adults, and since it has been shown that NWB possesses higher noise levels than laboratory platforms, and that noise levels increase with lower weight [15], we could expect less accurate measurement of sway in children when using the NWB.

We developed a software program similar to that of a previous study [13] with the purpose of examining reproducibility and concurrent validity of the NWB in a population of children and adolescents. The objectives of the current study were (1) to investigate reproducibility of the NWB and a laboratory force platform (AMTI) in a field setting, and (2) to explore the concurrent validity of the NWB when compared to the AMTI, in a field setting to test bilateral and unilateral balance in a random selection of children and adolescents.

## Methods

The current study is a substudy of “The Childhood Health Activity and Motor Performance School study – Denmark” (CHAMPS-Study DK). The CHAMPS-Study DK is a longitudinal cohort study [19] from August 2008 to July 2014 that includes 1300 participants from 10 public schools. The study is situated in the municipality of Svendborg, in the southern part of Denmark. Ethical approval was obtained for the CHAMPS-Study DK (project ID S-20080047). The study conforms with the declaration of Helsinki [20] and all parents have given written informed consent for their child to participate in the study.

### Participants

The participants for the current study were recruited from schools participating in the CHAMPS-Study DK. A random sample of 58 participants from the fourth, fifth and seventh grade (aged 10–14 years old) agreed to participate. Exclusion criteria were severe leg and back injuries or pain that would prevent the child from standing on one leg, illness (i.e., fever) during the last week, neurological disease and one or more missing follow-up measurements.

### Test procedure

The participants were tested in pairs, with one participant on the NWB and the other on the AMTI, with a randomized test order between the two platforms.

The sway tests were selected on the basis of their varying difficulty, suitability for the age group and common use [12,13,21]. Duration of each trial was 30 seconds, in line with previous studies [12,13,22,23].

Three rounds of four different sway tests were performed on both the NWB and the AMTI: bilateral stance with eyes open (1), unilateral stance on both dominant (2) and non-dominant leg (3) with eyes open, and bilateral stance with eyes closed (4). The dominant leg was defined as that used to kick a ball. The participant paused for 30 seconds between each round. After completion of three successful rounds on one platform, the participant had a break for 10–12 minutes before repeating the full procedure on the other platform.

Reproducibility was assessed using an intra-day test-retest design with a two-hour break between sessions. To ensure a stable physical state between sessions, none of the participants had physical education lessons previous to the sessions.

During all four tests, the participant was instructed to stand barefoot with their hands on their hips and to remain as calm as possible for the full duration of the test.

The bilateral stance was performed with feet together, heel to heel, toe to toe, and standing on the middle of the platform on a clearly marked cross to ensure a reproducible foot position throughout the data collection. The unilateral stance was performed standing with the middle of the tested foot on the cross, with the foot of the non-weight bearing leg placed in a resting, non-supporting position. During these tests, failure was defined as touchdown by one foot on the measurement equipment or on the floor. In case of failure, participants were allowed a new trial, with a maximum of three unsuccessful attempts per test.

The clinicians performing the tests were thoroughly instructed in all test procedures during a full day of practice that included standardized calibration of the equipment, and measurement and instruction procedures. The same clinician tested all participants on the AMTI, and the same two clinicians tested all participants on the NWB.

### Equipment and data

All measurements were performed in the school’s sports hall. The measurement tools were the NWB, a peripheral of the Wii gaming system (Nintendo Inc.), and the AMTI force platform (OR6-7-1000 with amplifier (MiniAmp MSA-6), Advanced Technologies, MA, USA). The AMTI and the NWB were placed exactly 2 meters from a solid wall, the AMTI facing one wall and the NWB facing another wall. A clearly marked cross was placed for visual fixation on the wall in front of each platform, 1.5 meters above floor level. The platforms were calibrated according to the manufacturer’s instructions, i.e. the AMTI before each test, and the NWB after each pause and every time the test equipment had been shut off.

The NWB (52.1 cm x 33.7 cm) was interfaced with a laptop computer using a custom-written Microsoft Windows application in accordance with a similar study [13], using the open-source library WiiMoteLib running under Windows 7 to access the Wii-data through a Bluetooth connection. The sampling rate was 60 Hz.

Data from the AMTI platform (50.8 cm × 46.4 cm) were amplified and digitized (National instrument A/D card) with a sample rate of 125 Hz. To reduce high-frequency noise from the NWB and AMTI data, a Butterworth low-pass filter with a cut-off value of 10 Hz was applied.

As COPL is considered analogous to the COP velocity when the trials have a fixed time interval, and to be comparable with previous studies [13], COPL was chosen as the outcome variable.

### Statistical analysis

The primary outcome used for the analysis of reproducibility and concurrent validity was the median of the three COPL measures from three successful trials in each of the four different tests. The median was chosen as it was used in a similar study [13], but also to eliminate possible outliers. Histograms and quantile-quantile plots were made to check the assumptions of normal distribution of COPL data and differences in COPL.

To quantify reproducibility of the measurement devices and the concurrent validity, Bland-Altman plots with 95% limits of agreement (LOA) were calculated [24]. Using the Bland-Altman plot the mean values from the two measurements are plotted on the mean differences from the two measurements, the plots should be centred around the line of zero difference. The 95% LOA presents the interval containing 95% of the plots and therefore visualizes the spread of the current measurements. Further, the Bland-Altman plots with the 95% LOA indicate systematic differences [25]. To quantitatively describe the intra-subject variability between sessions the standard error of measurement (SEM) and the minimum detectable change (MDC) were calculated. SEM was calculated as the standard deviation (SD) of the mean differences between test and retest divided by √2. MDC defines the limits within a change in the measurement score that could be attributed to measurement error. MDC is closely related to SEM as MDC is calculated as 1,96*√2*SEM [26]. MDC is also related to limits of agreement, as a true change in measure is only statistically significant and not due to measurement error, if the change in measure is outside the 95% LOA [27]. The percentage difference from the mean value was calculated as MDC/COPL mean * 100.

Coefficients of reproducibility and concurrent validity were assessed by the concordance correlation coefficient (CCC). The CCC assesses reliability as well as the ICC does [28,29], results in coefficients close to the ICC [28,29], and has also been found to be easy to use and interpret [29]. In the analysis of concurrent validity, CCC was calculated on the first session of tests for both platforms. Interpretations of CCC or ICC point estimates are not yet agreed upon [14,30-32]. In the current study CCC point estimates *≥ 0.70* were interpreted as satisfactory.

All calculations and statistical analysis were conducted in STATA (version 13.0) (Statacorp, College Station, Texas, USA).

## Results

Two participants were excluded due to ankle injuries and a further two were excluded because they could not complete the tasks according to the instructions. The 54 participants (45% boys) had a mean age of 11.5 years (range 10–14), mean height of 154.7 cm (SD 9.9 cm) and mean weight of 44.1 kg (SD 10.3 kg). The differences in number of participants in the reproducibility and concurrent validity analysis are due to missing in the follow up measurements.

### Test-retest reproducibility of NWB and AMTI

**Figure 1:**
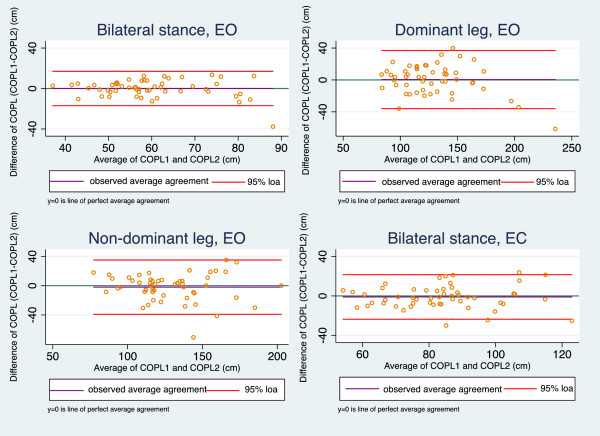
**Bland-Altman plots of reproducibility of the Nintendo Wii Board.** EO = Eyes Open, EC = Eyes Closed, 95% loa = 95% limits of agreement, COPL: Centre Of Pressure path Length, COPL1 = COPL test, COPL2 = COPL re-test.

Regarding the NWB, Bland-Altman plots of the average COPL (Figure 1) demonstrated no systematic bias. The line of observed agreement was approximately similar to the line of perfect agreement. The range of LOA was largest in the test for the dominant leg (Table 1).

**Table 1:** Test-retest reproducibility of centre of pressure path length during four different tests of static balance

	**n NWB/AMTI**	**Mean COPL (cm (SD)) NWB/AMTI**	**Mean COP velocity (cm (SD)) NWB/AMTI**	**CCC (95% CI)**	**Mean diff (cm (SD))**	**LOA (cm)**	**SEM (cm)**	**MDC (cm (%))**
**Bilat., EO**								
COPL1	54/52	59.7 (13.4)/53.7 (12.3)	1.99 (0.4)/1.79 (0.4)					
COPL2		59.9 (13.9)/51.9 (11.4)	1.99 (0.4)/1.73 (0.4)					
NWB				0.76 (0.65-0.87)	0.02 (8.7)	-17.0-17.0	6.1	16.9 (28.2)
AMTI				0.79 (0.69-0.89)	1.9 (7.5)	-12.8-16.5	5.3	14.7 (27.3)
**Dom. leg, EO**								
COPL1	53/51	129.3 (29.1)/135.2 (34.3)	4.31 (1.0)/4.51 (1.1)					
COPL2		128.9 (34.9)/133.2 (33.8)	4.30 (1.2)/4.44 (1.1)					
NWB				0.83 (0.75-0.91)	0.5 (18.6)	-35.8-36.7	13.1	36.3 (28.1)
AMTI				0.86 0.78-0.93)	2.0 (18.2)	-33.6-37.6	12.8	35.6 (26.3)
**Non-dom. leg, EO**								
COPL1	52/51	127.6 (27.3)/137.4 (31.7)	4.25 (0.9)/4.58 (0.7)					
COPL2		128.8 (29.5)/130.5 (30.0)	4.29 (1.0)/4.35 (1.0)					
NWB				0.77 (0.66-0.88)	-2.0 (18.9)	-38.9-34.9	13.3	36.9 (28.6)
AMTI				0.80 (0.70-0.90)	6.9 (18.4)	-29.2-43.0	13.0	36.1 (26.3)
**Bilat. EC**								
COPL1	53/52	83.7 (16.8)/76.1 (19.5)	2.76 (0.6)/2.54 (0.7)					
COPL2		84.6 (16.5)/74.5 (18.7)	2.81 (0.5)/2.48 (0.6)					
NWB				0.76 (0.65-0.88)	-1.0 (11.5)	-23.5–21.4	8.1	22.4 (26.5)
AMTI				0.83 (0.75-0.92)	1.7 (11.1)	-19.9-23.2	7.7	21.5 (28.1)

NWB: Nintendo Wii Board. AMTI: AMTI force platform, Bilat: bilateral stance, Dom: dominant, Non-dom: non-dominant, COPL: Centre Of Pressure path Length, SD: Standard Deviation, COP speed: Centre of pressure velocity, CCC: Concordance Correlation Coefficient, CI: Confidence Interval, Mean diff: Mean difference of the means, LOA: Limits of Agreement, SEM: Standard Error of the Measurement, MDC: Minimal Detectable Change, EO = eyes open, EC = eyes closed, COPL1 = COPL test, COPL2 = COPL re-test.

**Figure 2:**
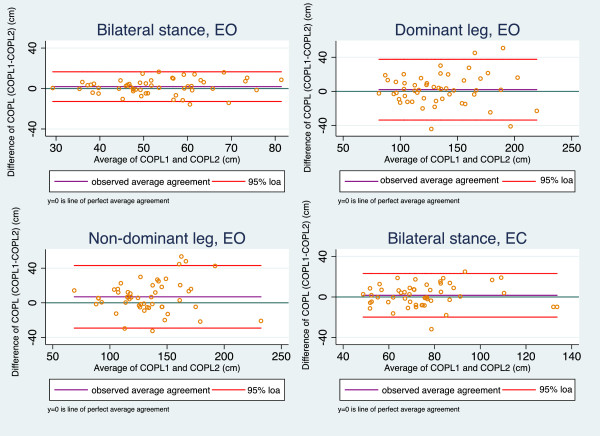
**Bland-Altman plots of reproducibility of the AMTI force platform.** EO = Eyes Open, EC = Eyes Closed, 95% loa = 95% limits of agreement, COPL: Centre Of Pressure path Length, COPL1 = COPL test, COPL2 = COPL re-test.

For the NWB, the CCC was ≥ 0.70, ranging from 0.76 to 0.83 (Table 1). The MDC varied between 16.9 and 36.9 cm (26.5-28.6% of the mean COPL). The mean COPL difference was highest for the unilateral test on the non-dominant leg.For the AMTI, Bland-Altman plots (Figure 2) demonstrated no systematic bias in three of the four tests. In the unilateral test on the non-dominant leg, however, the differences increased with larger values, and the observed agreement indicated longer COPL on retests. The range of LOA was largest in the test for the dominant leg.

For the AMTI, CCC values for COPL were ≥ 0.70 in all four tests, ranging from 0.79 to 0.86 (Table 1). MDC varied between 14.7 and 36.1 cm (26.3-28.1% of the mean COPL). The highest mean differences were seen in the unilateral tests.

In summary, among the eight Bland-Altman plots only one revealed a systematic bias (AMTI, unilateral test on the non-dominant leg), the CCC coefficients were slightly higher in AMTI, whereas MDC and LOA were comparable for the NWB and AMTI.

### Concurrent validity

**Figure 3:**
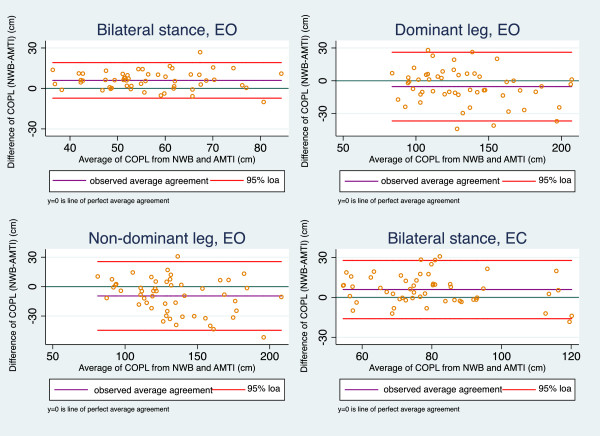
**Bland-Altman plots of concurrent validity, Nintendo Wii Board and AMTI force platform.** EO = Eyes Open, EC = Eyes Closed, 95% loa = 95% limits of agreement, COPL: Centre Of Pressure path Length, COPL1 = COPL test, COPL2 = COPL re-test.

Bland-Altman plots (Figure 3) demonstrated no systematic bias, except for the unilateral test on the non-dominant leg that showed a slight funnel effect, with larger differences between the two measurement devices as the sway measures increased. LOA showed larger variation in the unilateral tests than in the bilateral tests and the line of observed agreement indicated that the NWB gave longer measurements in bilateral tests, but shorter measurements in unilateral tests (Figure 3).

Point estimates of concurrent validity were satisfactory (CCC = 0.74-0.87) (Table 2). The mean difference was highest for the unilateral test on the non-dominant leg.

**Table 2:** Concurrent validity

**(n = 53)**	**CCC (95% CI)**	**SEM (cm)**	**Mean diff (cm (SD))**	**95% Limits of agreement (cm)**
Bilateral stance, EO	0.74 (0.63-0.85)	4.8	5.96 (6.73)	-7.2 – 19.1
Dominant leg	0.87 (0.81-0.94)	11.3	-5.33 (16.0)	-36.7 – 26.1
Non-dominant leg	0.79 (0.70-0.89)	12.6	-9.5 (17.9)	-44.5 – 25.5
Bilateral stance, EC	0.77 (0.66-0.87)	7.9	5.99 (11.17)	-16.0 – 27.8

Comparison of COPL using Nintendo Wii Board and AMTI force platform.

COPL: Centre Of Pressure path Length, CCC: Concordance Correlation Coefficient, 95% CI: 95% Confidence Interval, SEM: Standard Error of the Mean, Mean diff: Mean difference of the means, SD = Standard Deviation, EO = eyes open, EC = eyes closed.

Overall, both the 95% LOA and the CCC coefficients in the validity study were comparable to the results from the test-retest study.

## Discussion

The main findings of this study were that NWB is a reproducible and valid tool for measuring sway of children in a field setting, and that NWB and AMTI possess almost equal reproducibility of COPL in children (based on 95% LOA, MDC and CCC > 0,70), the AMTI presenting a slight tendency of systematic bias in the reproducibility study. Furthermore, a possible measurement error in the validity of the NWB towards AMTI is small compared to the intra-subject variability, since 95% LOA and CCC of NWB when compared to the AMTI, is comparable to 95% LOA and CCC of the test-retest study.

As this was the first study to examine reproducibility and concurrent validity of the NWB in a population of children, comparisons of CCC estimates, MDC values and conclusions are made to studies of sway measures in adult populations. Comparisons of MDC values to previous studies are limited, as only few studies on COPL as a balance measure, reported MDC [33].

In line with previous studies [13,15,16,18], reproducibility and concurrent validity of the NWB were found to be satisfying. Bland-Altman plots illustrating the reproducibility of the NWB and AMTI showed almost similar COPL, confirmed by CCC > 0.70 (CCC 0.76-0.86). The MDC of NWB in percentage was relatively high (26-28%) in the current study, but in line with a previous study [13], and was similar to that of the AMTI. The relatively large LOA and MDC indicated large variation between trials, however, which questions the validity of the CCC. The importance of this variation in determining the appropriateness of using NWB and AMTI to measure sway is unknown, but needs examination in future studies as it might influence the usefulness of NWB and AMTI measures as predictor of injuries or risk of falls. The NWB was found sensitive enough to detect postural changes associated with subtle variations in visual tasks in elderly people [17], and despite the indication of systematic bias in one test of the AMTI, the AMTI has shown to be sensitive enough to predict injuries from sway measures [4].

In previous test-retest studies on sway variables, the time interval between test and retest varied from a few minutes to several days and seemed to be arbitrarily chosen [11-13,23,34]. Previous studies have concluded that time of day may influence sway measures [35,36]. In the current timespan of two hours, Bland-Altman plots and reliability coefficients showed satisfactory test-retest, but more studies are needed to examine time-of-day and day-to-day variation in sway measures among children and adolescents.

Overall, the reproducibility of the NWB and the AMTI was satisfactory. The impact of the intra-subject variability on the precision and feasibility of the equipment when used in clinical settings and field studies is yet to be examined.

The validity of NWB is difficult to assess directly, but by comparing LOA from the validity study with LOA in the reproducibility study it is possible to have an indication of the size of measurement error of the NWB. As the LOA and CCC coefficients in the reproducibility study are comparable to the LOA and the coefficients in the validity study, the measurement error due the NWB is probably small compared to the intra-subject variability. CCC for COPL was satisfactory (CCC 0.74-0.86). Thus, if the variable of interest is COPL, the results for the NWB are comparable to those for the AMTI, confirming previous studies [13,18]. In favour of NWB is further, that it is economically feasible to measure sway in large populations, due to the small size, light weight, and that it is easy to use and cheap compared to advanced instruments.

The cut-off point for interpreting CCC values was chosen because of similarities in interpretation between CCC and ICC [28,29], and to make this field study in a child population comparable with the two other studies that evaluated both concurrent validity and reproducibility of the NWB [13,18]. Although cut-off points for interpreting the ICC value are not yet agreed upon [14,30-32], the agreement of all four tests is convincing when the variable of interest is COPL.

Findings of a satisfactory reproducibility for the NWB and level of agreement with the AMTI platform are in line with adult studies [13,15,16,18]. However, the CCC of all tests in the present study was generally lower. The reason for these differences is not known, but may be due to lack of full motor control development and less secure balance among the participants [6-9], resulting in more variation between test and retest. The ability to focus on the task may also have been an issue compared to the selected adults recruited for the study by Clark et al. [13]. These issues, along with intra-subject variability, are important in determining which age groups the sway measurements are relevant for, and especially whether they are relevant for children who are younger than the participants in the current study.

Bland-Altman plots of concurrent validity revealed that NWB seemed to produce longer COPL measures in bilateral tests and shorter COPL measures in the unilateral tests than the AMTI, indicating systematic bias. However, as the differences between NWB and AMTI were small and the CCC coefficients from the validity analysis were satisfactory, we consider this issue to be of minor importance.

We found higher SEM, mean differences and larger LOA in the unilateral tests than in the bilateral tests, both in the test-retest analysis and in the validity analysis. This difference is mainly ascribed to an anticipated higher level of difficulty due to the smaller medial-lateral base of support area in single-leg tests compared to bilateral tests. The performance of the participants will be more homogeneous in the bilateral tests, since variation between the trials is smaller. However, in some populations there may be floor effects when using only bilateral balance tests.

The use of NWB as a tool to measure sway, and the comparison of NWB with an AMTI platform has been debated by e.g. Pagnacco et al. [37] because of too much noise in the NWB measures, when it was compared to a platform manufactured by Pagnacco, and because the AMTI measures not only COPL but also three-dimensional measures as rambling and trembling. However, in the current study, since comparisons of the NWB with AMTI was only made to the COPL measures, and the noise of measurement primarily was found to be due to intra-subject variability, the indicated differences seem to be of minor importance. Overall the concurrent validity of the NWB was satisfactory when compared to the AMTI. The impact of the intra-subject variability on the precision and feasibility of the equipment when used in clinical settings, field studies and studies of injury prediction are yet to be examined, as is the reproducibility of NWB and AMTI in children younger than the current study population.

### Limitations

A limitation of the current study is the lack of dynamic tests. The static balance is only one component of balance [6,10], and therefore the results in the current study cannot be generalized to measures of the total concept of balance.

The validity study was performed with two single measures of each sway platform, and not by putting NWB on top of the AMTI as seen in a previous study by Huurnink et al. [15]. Although we tried to take intra-subject variability into account in the discussion of the results, the current method is probably not able to detect small systematic errors, and the possibility of an unknown sized bias of NWB measurements remains.

The analyses were not stratified by age and sex. In the test-retest analysis, we did post hoc analysis stratified by grade and sex. We found higher CCC values in the unilateral tests for participants in the 7^th^ grade compared to the 4^th^ grade. This supports the previously mentioned theory of age-specific differences due to lack of motor control development and reduced ability to focus. Furthermore, CCC values for boys were lower than those for girls in all tests. Even though these values could be biased due to small sample size, there is a need for future studies to look into differences in sway performance between age groups and genders. It seems important to consider age and gender when selecting subjects for testing.

### Strengths

The main strength of the current study is its field setting. It was not possible to avoid all disturbances during the tests as the children were curious to see what was going on, and there were also soccer and playground activities outside the sports hall. In spite of this, however, the results from the reproducibility and validity analyses were satisfactory.

The high degree of feasibility, makes it possible to use the NWB not only as a measurement tool in sports clinics, but also as a new tool to use in field studies and larger cohort studies, with the need for an objective measure of a static balance component. The inclusion of both unilateral and bilateral balance tests, and the large numbers of participants, are also strengths of the current study.

## Conclusion

NWB and AMTI both have satisfactory reproducibility for bilateral and unilateral static balance tests in a child population. Concurrent validity of the NWB was satisfactory when compared to the AMTI. The NWB appears to be a reliable and valid low-cost tool that could replace the AMTI in field settings and in larger cohort studies including children. Future studies are needed to examine intra-subject variability and to test the predictive validity of NWB in a child population.

## Abbreviations

COPL: Centre of pressure path length; COP: Centre of pressure; NWB: Nintendo wii board; AMTI: AMTI laboratory force platform; CHAMPS-Study DK: “The childhood health activity and motor performance school study–Denmark”; ICC: Intra class correlation; CCC: Concordance correlation coefficient; LOA: Limits of agreement; SD: Standard deviation; SEM: Standard error of measurement; MDC: Minimum detectable change.

## Competing interests

The authors declare that they have no competing interests.

## Authors’ contributions

Authors’ contributions were as follows: LRL, NW, MGJ, TJ, and BJK contributed to the design of the study. LRL collected the data. LRL, NW and BJK performed the data management. LRL and NW performed the data analysis and LRL, NW and BJK were in charge of data interpretation. LR and MGJ wrote the manuscript. All authors participated in data interpretation and contributed to manuscript revision, and each author vouches for the integrity of the work.

## Pre-publication history

The pre-publication history for this paper can be accessed here:

http://www.biomedcentral.com/1471-2431/14/144/prepub
